# Synthesis of *C*_2_-Symmetric Benzimidazolium Salts and Their Application in Palladium-Catalyzed Enantioselective Intramolecular α-Arylation of Amides

**DOI:** 10.3390/molecules21060742

**Published:** 2016-06-08

**Authors:** Weiping He, Wei Zhao, Bihui Zhou, Haifeng Liu, Xiangrong Li, Linlin Li, Jie Li, Jianyou Shi

**Affiliations:** 1 School of Medicine, Zhejiang University City College, No. 48, Huzhou Road, Hangzhou 310015, China; wphe2016@163.com (W.H.); zhaowei@zucc.edu.cn (W.Z.); 18868816170@163.com (B.Z.); maplewandering@126.com (H.L.); lixr@zucc.edu.cn (X.L.); lill@zucc.edu.cn (L.L.); 2 Individualized Medication Key Laboratory of Sichuan Province, Sichuan Academy of Medical Science & Sichuan Provincial People’s Hospital, School of Medicine, Center for Information in Medicine, University of Electronic Science and Technology of China, Chengdu 610072, China; 3 College of Pharmaceutical Sciences, Zhejiang University, Hangzhou 310058, China

**Keywords:** *N*-heterocyclic carbene, benzimidazolium, Pd-catalyzed, asymmetric intramolecular α-arylation

## Abstract

A series of *C*_2_-symmetric chiral benzimidazolium salts, the precursor of *N*-heterocyclic carbene ligands, were designed and synthesized from 1,2-dibromobenzene. *In situ* prepared corresponding carbenes were tested in the asymmetric palladium-catalyzed intramolecular α-arylation of amides, affording chiral diarylmethanols with high yields and moderate enantioselectivities.

## 1. Introduction

Oxindoles (=1,3-dihydro-2*H*-indol-2-ones) bearing a quaternary stereogenic center at the C (3) position represent a prominent structural motif in many natural products and biologically active compounds [1,2,3,4,5,6], and the development of synthetic methods for these compounds is of great importance in organic chemistry. Consequently, asymmetric transition metal-catalyzed reactions that provide access to enantiomerically enriched 3-alkyl-3-aryl oxindoles were established over the past decade: Overman’s elegant intramolecular Heck reactions [7,8,9], Trost’s Pd- or Mo-mediated allylic alkylations [10,11,12], and the Pd-catalyzed intramolecular α-arylation of amides, which are the focus of the present study.

**Scheme 1:**
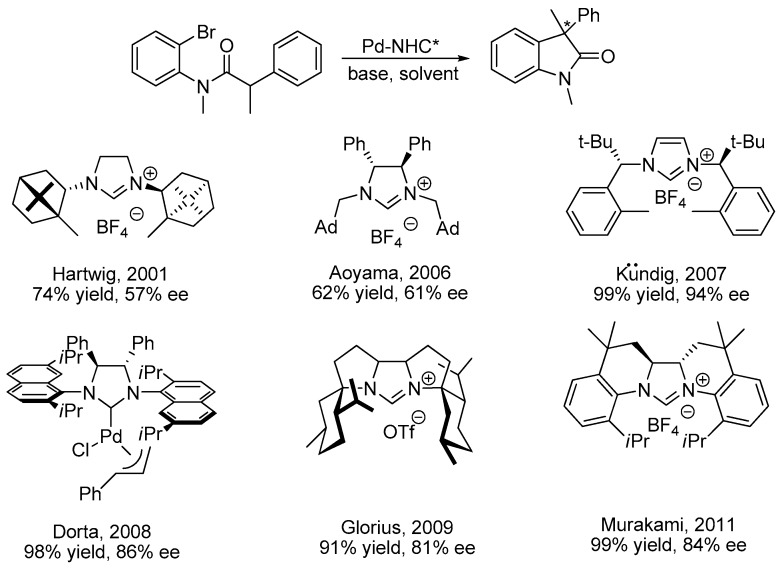
Representative ligands in Pd-catalyzed asymmetric intramolecular arylation of amides.

Pioneered by Hartwig and co-workers, the intramolecular α-arylation of amides provides efficient and direct access to chiral 3,3-disubstituted oxindoles. Bulky chiral *N*-heterocyclic carbene (NHC) ligands worked best for the asymmetric transformation (up to 70% ee) [13]. This study was followed by those of the groups of Glorius and Aoyama, but only moderate ee values were obtained [14,15,16]. A significant improvement in this Pd-catalyzed asymmetric reaction was achieved by Kündig and co-workers [17,18,19,20,21]. Since then, this chemistry has been expanded further and several other chiral carbene ligands have been reported to give the desired product in excellent enantioselectivities (Scheme 1). Dorta and co-workers reported new NHC ligands with chiral *N*-heterocycle and naphthyl side chains and their successful application in a Pd-catalyzed asymmetric reaction. A series of 3-alkyl-3-aryl [22], 3-allyl-3-aryl [23], and 3-flouro-3-aryl [24] oxindoles were synthesized. Additionally, conformationally restricted chiral ligands developed by Glorius [25] and Murakami [26] also showed high asymmetric induction in this reaction. Despite the successes in this field, new efficient chiral NHC ligands for this reaction are still needed. In this paper, we would like to report our investigation on the enantioselective intramolecular α-arylation of amide with the new chiral carbene ligands incorporating the benzimidazole skeleton (Scheme 1).

## 2. Results

**Scheme 2:**
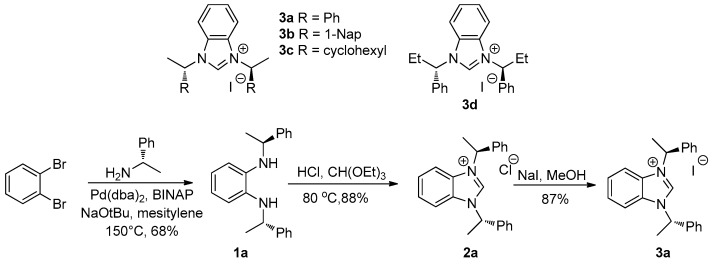
Representative synthesis of benzimidazolium salt.

The synthesis of the benzimidazolium salt **3a** as an *N*-heterocyclic carbene precursor is representatively shown in Scheme 2. Buchwald-Hartwig coupling of 1,2-dibromobenzene with (*S*)-α-methylbenzylamine gave the disubstituted product **1a** in 80% yield. Next, treatment of diamine with HCl in CH(OEt)_3_ gave the benzimidazolium salt **2a** in 85% yield. The hygroscopic chloride salt **2a** which became gel on exposure to the atmosphere was difficult to handle on the benchtop, but this problem was solved by anion metathesis with NaI to give **3a**. Other benzimidazolium salts **3b**–**d** were prepared in the same manner. All of the benzimidazolium salts **3a**–**d** were purified and fully characterized by NMR and mass spectrometry. Furthermore, this method works equally well for milligram and multigram quantities.

With the new chiral benzimidazolium salts in hand, we turned our attention to their application in the Pd-catalyzed asymmetric intramolecular arylation of amides. Ligand precursors **3a**–**d** were tested in the intramolecular α-arylation of **4a** following Hartwig’s *in situ* method (Table 1) [13]. Among the benzimidazolium salts screened, **3c** possessing a cyclohexyl group as the R substituent gave better asymmetric induction (40% ee, entry 3). With **3c** as an *N*-heterocyclic carbene ligand precursor, the use of other solvents such as 1,4-dioxane, toluene and THF gave less satisfactory results (entries 5–7). Other bases such as KO*^t^*Bu, LiO*^t^*Bu, KOH, and LiOH gave no better results than NaO*^t^*Bu (entries 8–11). Different palladium sources were also investigated with **3c**, and [Pd(allyl)Cl]_2_ emerged as the best choice of catalyst precursor (entry 14). Upon lowering the reaction temperature to rt, almost no reaction occurred; however, a 41% conversion and 48% ee were observed at 50 °C (Table 1, entries 16, 17).

**Figure 1:**
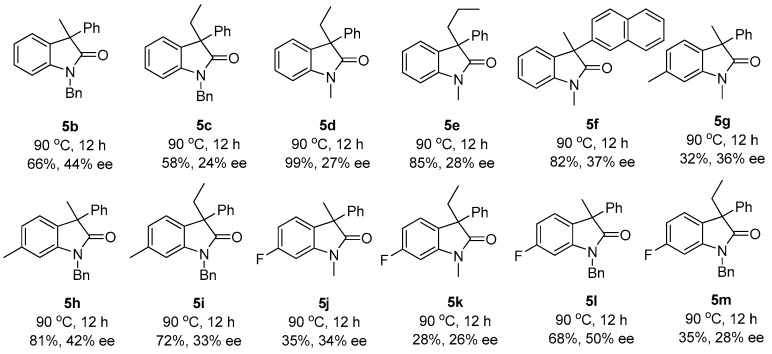
The asymmetric reaction products.

In the next step, different 2-bromoanilides were applied in the reaction with salt **3c** as a catalyst precursor. As shown in Figure 1, various substrates worked well with **3c** to give oxindoles in moderate to good yields (28%–99%), and the best ee value was up to 50%.

## 3. Experimental Section

### 3.1. General

MS spectra were measured on a Finnigan LCQDECA XP instrument and a Agilent Q-TOF 1290 LC/6224 MS system; ^1^H- and ^13^C-NMR spectra were obtained on Bruker AVANCE III 500 MHz and 600 MHz spectrometers (Bruker Co., Faellanden, Switzerland) with TMS as the internal standard; silica gel GF254 and H (10–40 mm, Qingdao Marine Chemical Factory, Qingdao, China) were used for TLC and CC. Unless otherwise noted, all reactions were carried out under an atmosphere of argon or nitrogen.

### 3.2. Procedure for the Synthesis of Compounds ***1a***–***d***

Pd_2_(dba)_3_ (73.3 mg, 0.08 mmol) and (±)-BINAP (99.6 mg, 0.16 mmol) were dissolved in mesitylene (10 mL) and the solution degassed for 15 min before being heated at 150 °C for 10 min (solution turns from deep purple to dark orange). Upon cooling sodium tert-butoxide (769 mg, 8.0 mmol), (*S*)-α-methylbenzylamine (1212 mg, 10.0 mmol) and 1,2-dibromobenzene (472 mg, 2.0 mmol) were added and the reaction mixture was heated to 150 °C for 16 h. The solution was allowed to cool and filtered through a pad of celite. Solvents were removed under reduced pressure and the crude material was purified by column chromatography eluting with light petroleum/ethyl acetate (50/1). Red oil (430 mg, 68%); ^1^H-NMR spectra of **1a** was identical to those reported in the literature [27].

Analogous compounds **1b**–**d** were prepared according to the similar procedure for **1a**. **1b**: 82% yield; ^1^H-NMR (500 MHz, CDCl_3_) δ: 8.31–7.39 (m, 14H), 6.41 (m, 4H), 5.35 (q, *J* = 6.4 Hz, 2H), 1.74 (t, *J* = 9.2 Hz, 6H). **1c**: 85% yield; ^1^H-NMR (500 MHz, CDCl_3_) δ: 6.84–6.48 (m, 4H), 3.41–3.20 (m, 2H), 1.90–0.98 (m, 28H). **1d**: 84% yield; ^1^H-NMR (500 MHz, CDCl_3_) δ: 7.31 (m, 10H), 6.51 (m, 4H), 4.25 (t, *J* = 6.5 Hz, 2H), 2.01–1.77 (m, 4H), 1.01 (t, *J* = 7.4 Hz, 6H).

### 3.3. Procedure for the Synthesis of Benzimidazolium Salts ***3a***–***d***

**1a** (411 mg, 1.3 mmol) was dissolved in 50 mL triethylorthoformate, then concentrated hydrochloric acid (37% *w*/*w*, 7.8 mmol, 656 µL of solution) was added at room temperature and the mixture was stirred for 30 min. Then the mixture was heated to 80 °C under air atmosphere for 12 h. After cooling to room temperature, ether (30 mL) was added. The precipitate was collected by filtration. The collected solids were dissolved in MeOH (10 mL) stirred with 5 equiv NaI at room temperature for 12 h. The collected solution was concentrated and the residue was allowed to react with NaI again. After evaporation of volatiles, the residue was purified by column chromatography (CH_2_Cl_2_/MeOH = 15/1) to give **3a** (454 mg, 77%). The ^1^H-NMR and HRESIMS spectra of **3a** were similar to those reported in the literature [27].

Analogous compounds **3b**–**d** were prepared according to the similar procedure for **3a**, HR-ESIMS, ^1^H- and ^13^C-NMR data see Supplementary Materials. **3b**: 80% yield; ${\left[\alpha \right]}_{\text{D}}^{20}$ = +157.8 (*c* 0.2, CH_2_Cl_2_); ^1^H-NMR (500 MHz, CDCl_3_) δ: 11.47 (s, 1H), 8.16–7.25 (m, 18H), 7.08 (q, *J* = 6.9 Hz, 2H), 2.51 (d, *J* = 6.9 Hz, 6H); ^13^C-NMR (125 MHz, CDCl_3_) δ: 141.13, 134.02, 132.47, 130.19, 129.52, 127.65, 126.95, 126.38, 125.48, 124.82, 121.76, 114.36, 77.29, 76.78, 56.13, 21.09; HR-ESIMS: *m*/*z* 427.2294 [M − I]^+^ (calcd for C_31_H_27_N_2_, 427.2169). **3c**: 83% yield; ${\left[\alpha \right]}_{\text{D}}^{20}$ = +0.5 (*c* 0.2, CH_2_Cl_2_); ^1^H-NMR (500 MHz, CDCl_3_) δ: 11.23 (s, 1H), 7.71 (m, 4H), 4.91–4.81 (m, 2H), 2.51–1.75 (m, 14H), 1.47–0.77 (m, 14H); ^13^C-NMR (125 MHz, CDCl_3_) δ: 141.29, 131.06, 126.91, 114.33, 77.30, 76.79, 61.42, 42.47, 29.69, 29.45, 25.66, 25.55, 25.49, 18.32; HR-ESIMS: *m*/*z* 339.3016 [M − I]^+^ (calcd for C_23_H_35_N_2_, 339.2795). **3d**: 81% yield; ${\left[\alpha \right]}_{\text{D}}^{20}$ = −19.5 (*c* 0.2, CH_2_Cl_2_); ^1^H-NMR (500 MHz, CDCl_3_) δ: 11.82 (s, 1H), 7.66–7.32 (m, 14H), 6.03 (t, *J* = 7.9 Hz, 2H), 2.88–2.74 (m, 4H), 1.07 (t, *J* = 7.3 Hz, 6H); ^13^C-NMR (125 MHz, CDCl_3_) δ: 136.21, 131.05, 129.45, 129.27, 127.40, 127.08, 114.41, 77.29, 76.78, 65.16, 27.11, 11.04; HR-ESIMS: *m*/*z* 355.2381 [M − I]^+^ (calcd for C_25_H_27_N_2_, 355.2169).

### 3.4. Representative Procedure for the Pd-Catalyzed Intramolecular α-Arylation of Amides

Pd_2_(dba)_3_ (4.6 mg, 0.005 mmol), chiral benzimidazolium iodide **3c** (carbene ligand precursor) (4.7 mg, 0.01 mmol) and sodium *tert*-butoxide (29 mg, 0.3 mmol) were placed under N_2_ in a dry Schlenk tube. Dimethoxyethane (DME) (0.05 M in substrate, freshly distilled over Na) was added and the mixture was stirred for 5 min. The 2-bromo-*N*-alkylanilide (0.2 mmol) was then added as a solution in DME (equal volume as above). The reaction was stirred at room temperature for 12 h. The reaction was treated with aq. NH_4_Cl (2 mL) and extracted with ether (3 × 2 mL). The combined organic phases were washed with water (3 mL) and brine (3 mL), and dried over Na_2_SO_4_. Flash chromatography afforded the product oxindoles. The enantiomeric purity of products **5a**–**m** was determined by chiral HPLC Analysis.

**5a**: 99% yield, 46% ee; The spectral data were comparable to those reported [17]. The ee was determined by HPLC analysis with Daicel Chiralcel OD-H (hexane/*i*-PrOH = 99/1, flow rate = 1.0 mL/min, tr (major) = 12.7 min, tr (minor) = 15.4 min); **5b**: 66% yield, 44% ee; The spectral data were comparable to those reported [17]. The ee was determined by HPLC analysis with Daicel Chiralcel OD-H (hexane/*i*-PrOH = 99/1, flow rate = 1.0 mL/min, tr (major) = 14.5 min, tr (minor) = 16.4 min); **5c**: 58% yield, 24% ee; The spectral data were comparable to those reported [25]. The ee was determined by HPLC analysis with Daicel Chiralcel OD-H (hexane/*i*-PrOH = 99/1, flow rate = 1.0 mL/min, tr (minor) = 13.7 min, tr (major) = 16.2 min); **5d**: 99% yield, 27% ee; The spectral data were comparable to those reported [21]. The ee was determined by HPLC analysis with Daicel Chiralcel OD-H (hexane/*i*-PrOH = 99/1, flow rate = 1.0 mL/min, tr (minor) = 11.3 min, tr (major) = 14.2 min); **5e**: 85% yield 28% ee; The spectral data were comparable to those reported [15]. The ee was determined by HPLC analysis with Daicel Chiralcel OD-H (hexane/*i*-PrOH = 99/1, flow rate = 1.0 mL/min, tr (minor) = 12.6 min, tr (major) = 15.7 min); **5f**: 82% yield, 28% ee; The ee was determined by HPLC analysis with Daciel Chiralcel OD-H (hexane/*i*-PrOH = 98/2, flow rate = 1.0 mL/min, tr (minor) = 15.4 min, tr (major) = 20.2 min); **5g**: 32% yield, 26% ee; The ee was determined by HPLC analysis with Daciel Chiralcel OD-H (hexane/*i*-PrOH = 99/1, flow rate = 1.0 mL/min, tr (major) = 10.6 min, tr (minor) = 12.2 min); **5h**: 81% yield, 42% ee; The ee was determined by HPLC analysis with Daciel Chiralcel OD-H (hexane/*i*-PrOH = 99/1, flow rate = 1.0 mL/min, tr (major) = 10.7 min, tr (minor) = 12.1 min); **5i**: 72% yield, 33% ee; The ee was determined by HPLC analysis with Daciel Chiralcel OD-H (hexane/*i*-PrOH = 99/1, flow rate = 1.0 mL/min, tr (minor) = 11.3 min, tr (major) = 12.2 min); **5j**: 35% yield, 34% ee; The ee was determined by HPLC analysis with Daciel Chiralcel OD-H (hexane/*i*-PrOH = 99/1, flow rate = 1.0 mL/min, tr (major) = 11.9 min, tr (minor) = 15.3 min); **5k**: 28% yield, 26% ee; The ee was determined by HPLC analysis with Daciel Chiralcel OD-H (hexane/*i*-PrOH = 99/1, flow rate = 1.0 mL/min, tr (minor) = 11.3 min, tr (major) = 12.7 min); **5l**: 68% yield, 50% ee; The ee was determined by HPLC analysis with Daciel Chiralcel OD-H (hexane/*i*-PrOH = 99/1, flow rate = 1.0 mL/min, tr (major) = 11.6 min, tr (minor) = 13.6 min); **5m**: 35% yield, 28% ee; The ee was determined by HPLC analysis with Daciel Chiralcel OD-H (hexane/*i*-PrOH = 99/1, flow rate = 1.0 mL/min, tr (minor) = 11.9 min, tr (major) = 13.2 min).

## 4. Conclusions

In conclusion, four chiral *C_2_*-symmetric benzimidazolium salts **3a**–**d** have been prepared. Their applicability in the Pd-catalyzed asymmetric intramolecular arylation of amides has been demonstrated, and the corresponding oxindoles were obtained with high yields and moderate enantiomeric excesses (up to 50%). Further application to other catalytic asymmetric reactions is now in progress.

## Figures and Tables

**Table 1:**
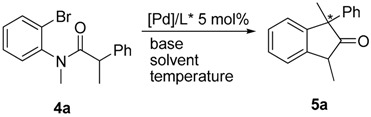
Chiral carbene ligands in the Pd-catalyzed intramolecular cylization of amide **4a** to oxindole **5a**.

Entry ^a^	Ligand	[Pd]	T (°C)	Solvent	Base	Yield (%) ^b^	ee (%) ^c^
1	**3a**	Pd_2_(dba)_3_	90	DME	NaO*^t^*Bu	51	14
2	**3b**	Pd_2_(dba)_3_	90	DME	NaO*^t^*Bu	63	9
3	**3c**	Pd_2_(dba)_3_	90	DME	NaO*^t^*Bu	98	40
4	**3d**	Pd_2_(dba)_3_	90	DME	NaO*^t^*Bu	95	9
5	**3c**	Pd_2_(dba)_3_	90	dioxane	NaO*^t^*Bu	98	25
6	**3c**	Pd_2_(dba)_3_	90	toluene	NaO*^t^*Bu	98	17
7	**3c**	Pd_2_(dba)_3_	90	THF	NaO*^t^*Bu	96	30
8	**3c**	Pd_2_(dba)_3_	90	DME	KO*^t^*Bu	72	25
9	**3c**	Pd_2_(dba)_3_	90	DME	LiO*^t^*Bu	−	−
10	**3c**	Pd_2_(dba)_3_	90	DME	KOH	99	38
11	**3c**	Pd_2_(dba)_3_	90	DME	LiOH	−	−
12	**3c**	Pd(OAc)_2_	90	DME	NaO*^t^*Bu	99	37
13	**3c**	PdCl_2_	90	DME	NaO*^t^*Bu	96	40
14	**3c**	[Pd(allyl)Cl]_2_	90	DME	NaO*^t^*Bu	99	46
15	**3c**	Pd[P(C_6_H_5_)_3_]_4_	90	DME	NaO*^t^*Bu	54	29
16	**3c**	[Pd(allyl)Cl]_2_	50	DME	NaO*^t^*Bu	41	48
17	**3c**	[Pd(allyl)Cl]_2_	rt	DME	NaO*^t^*Bu	trace	−

^a^ Reaction condition: [Pd] (5 mol %), ligand (5 mol %), base (1.5 equiv), 12 h; ^b^ Isolated yields; ^c^ Determined by chiral HPLC (CHIRALCEL OD Column) analysis.

## Supplementary Materials

Supplementary materials can be accessed at: http://www.mdpi.com/1420-3049/21/6/742/s1.
