# Supplementary material for: Synthesis of C2-Symmetric Benzimidazolium Salts and Their Application in Palladium-Catalyzed Enantioselective Intramolecular α-Arylation of Amides
Source: Molecules. 2016 Jun 8;21(6):742. doi: 10.3390/molecules21060742 (PMC6274209; doi:10.3390/molecules21060742)
Supplement: Supplementary file 1 [file molecules-21-00742-s001.pdf]

Supplementary Materials: Synthesis of C_2-Symmetric Benzimidazolium Salts and Their Application in Palladium-Catalyzed Enantioselective Intramolecular α-Arylation of Amides

Weiping He, Wei Zhao, Bihui Zhou, Haifeng Liu, Xiangrong Li, Linlin Li, Jie Li and Jianyou Shi

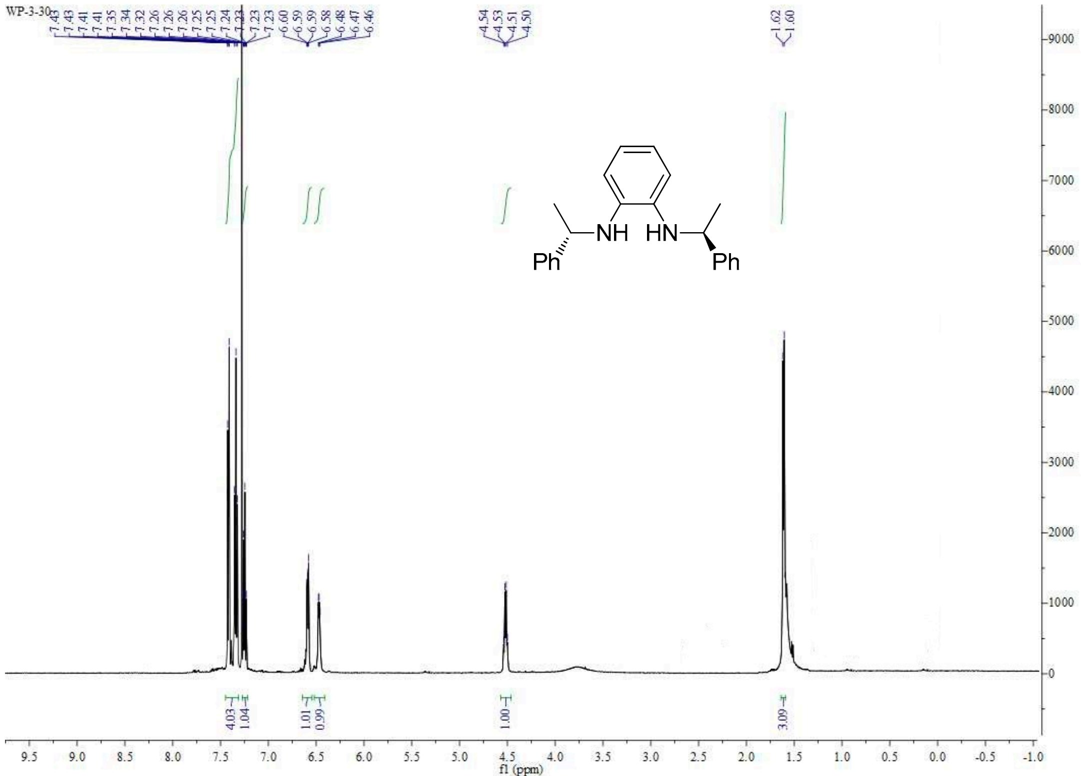

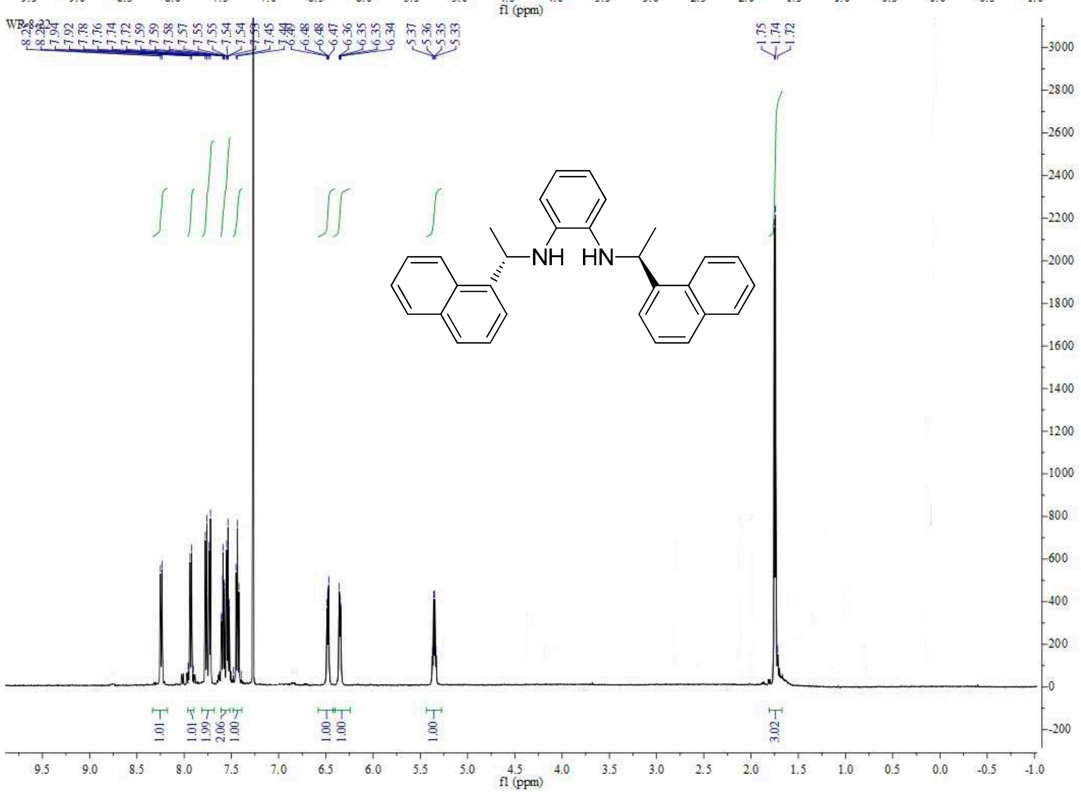

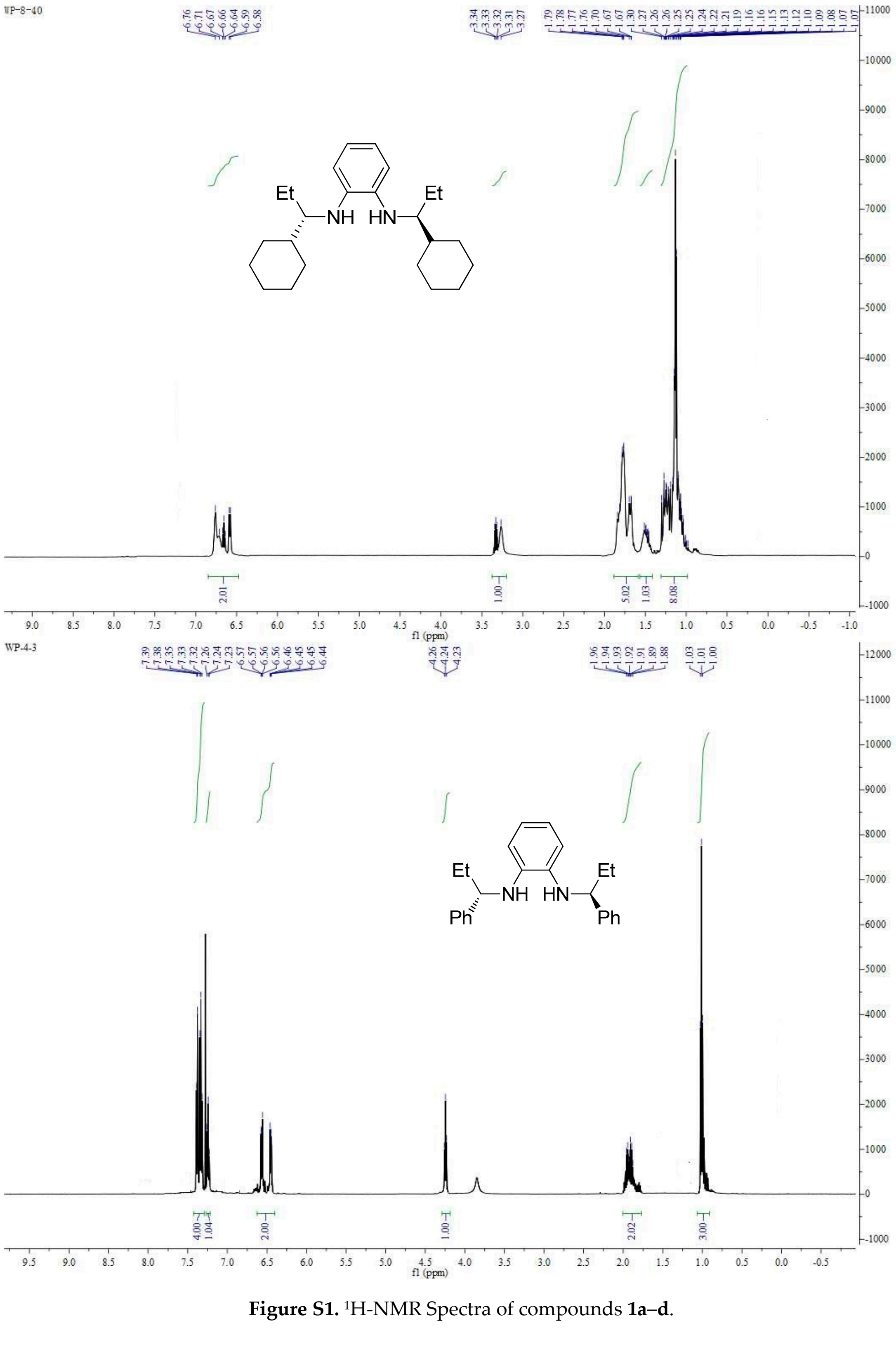

Figure S1. ^{1}H-NMR Spectra of compounds **1a–d**.

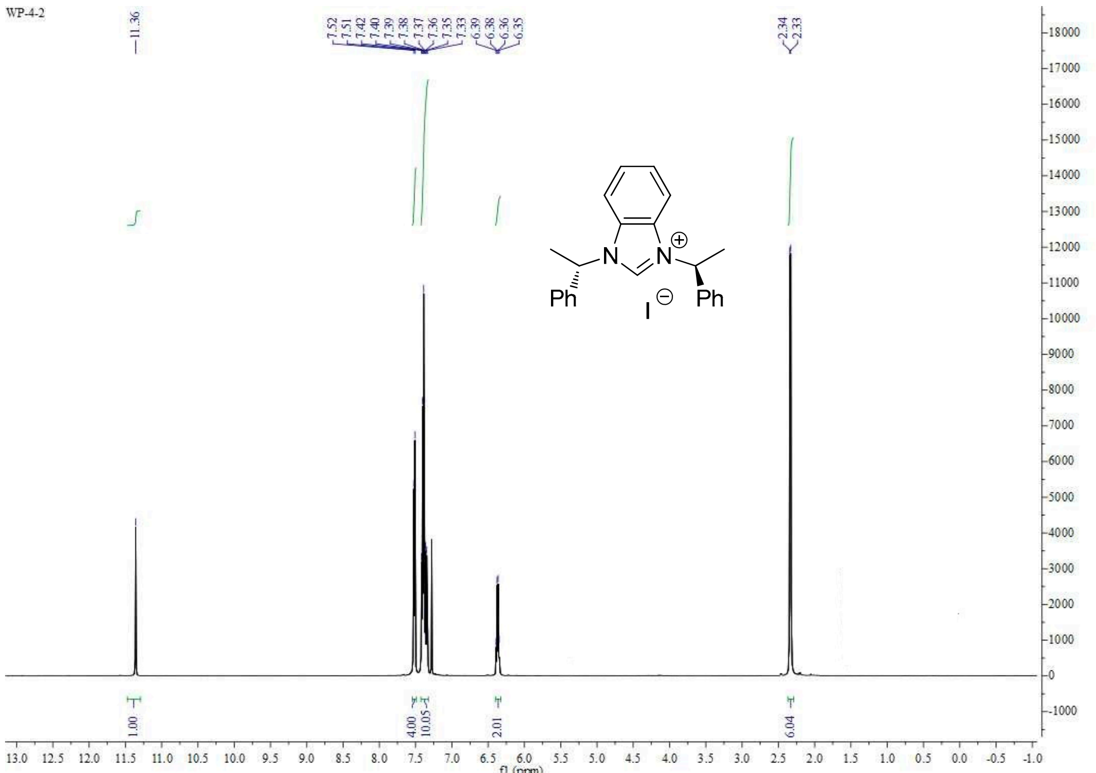
WP-4-2
11.36
7.52 7.51 7.42 7.40 7.39 7.38 7.37 7.36 7.35 7.33 6.39 6.38 6.36 6.35
2.34 2.33
1.00
4.00
10.05
2.01
6.04
Ph
Ph
N
N
I
f1 (ppm)

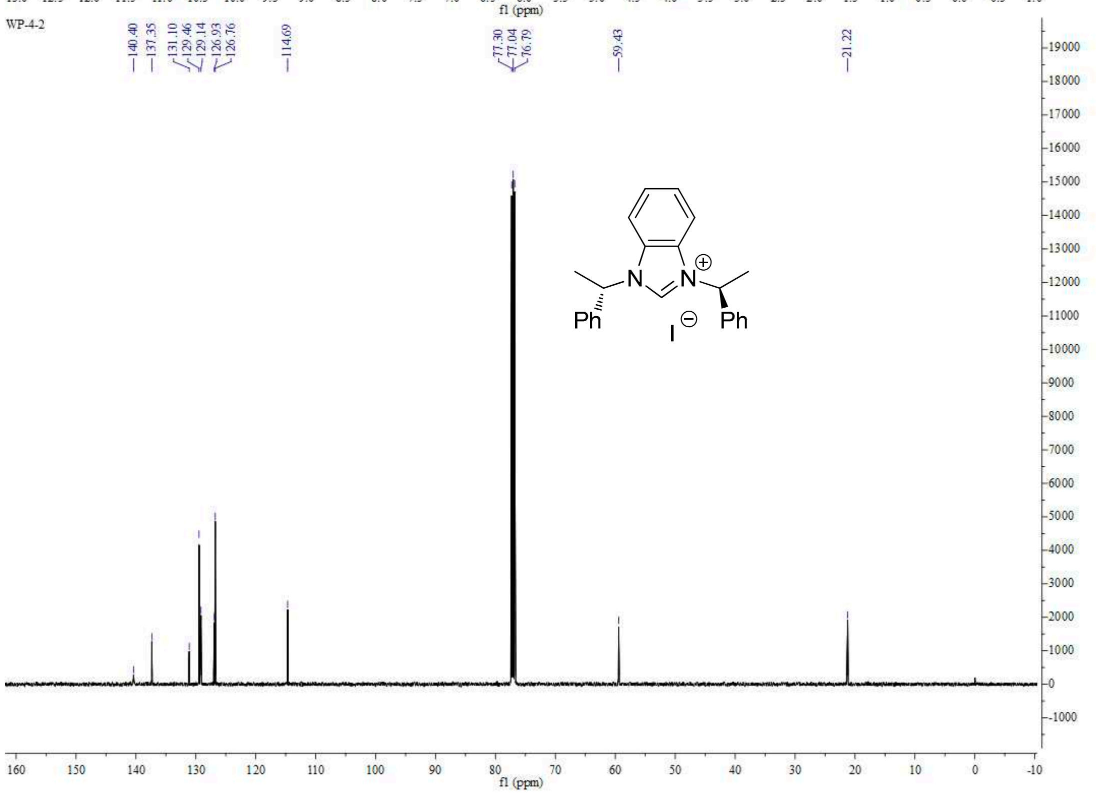
WP-4-2
140.40 137.35 131.10 129.46 129.14 126.93 126.76
114.69
77.30 77.04 76.79
59.43
21.22
Ph
Ph
N
N
I
f1 (ppm)

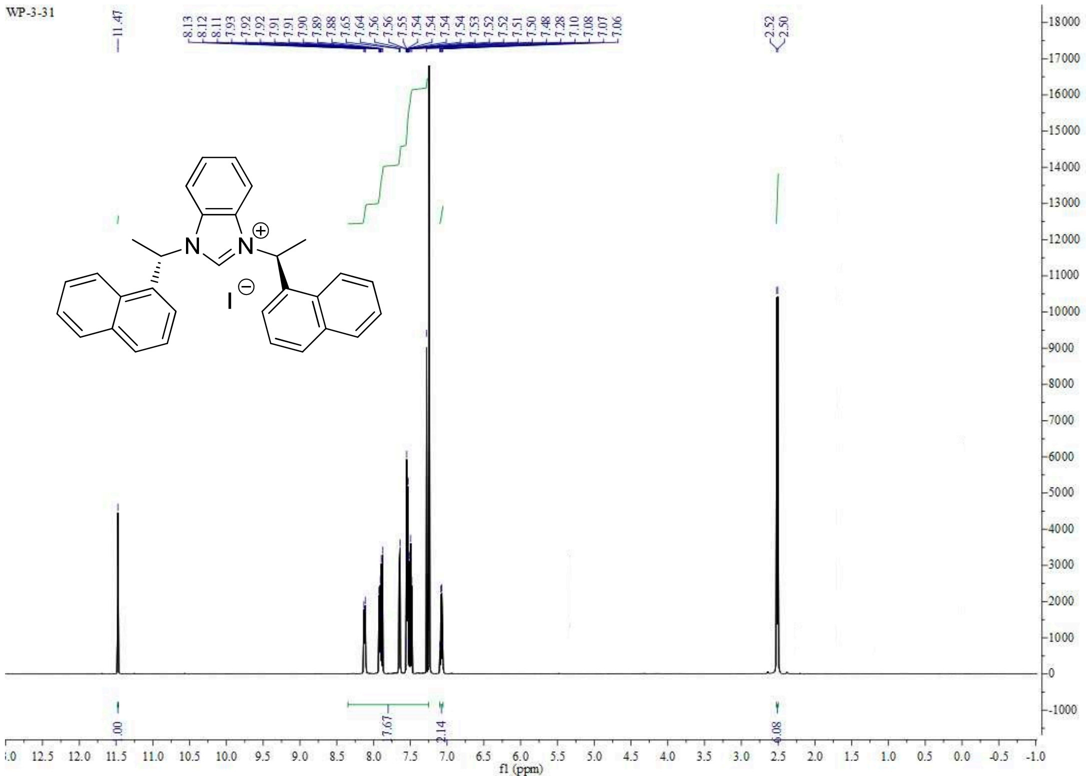
WP-3-31
11.47
8.13 8.12 8.11 7.93 7.92 7.92 7.91 7.91 7.90 7.89 7.88 7.65 7.64 7.56 7.56 7.55 7.54 7.54 7.54 7.54 7.53 7.52 7.52 7.51 7.50 7.48 7.28 7.10 7.08 7.07 7.06
2.52 2.50
N
N
I
.00
7.67
2.14
6.08
f1 (ppm)

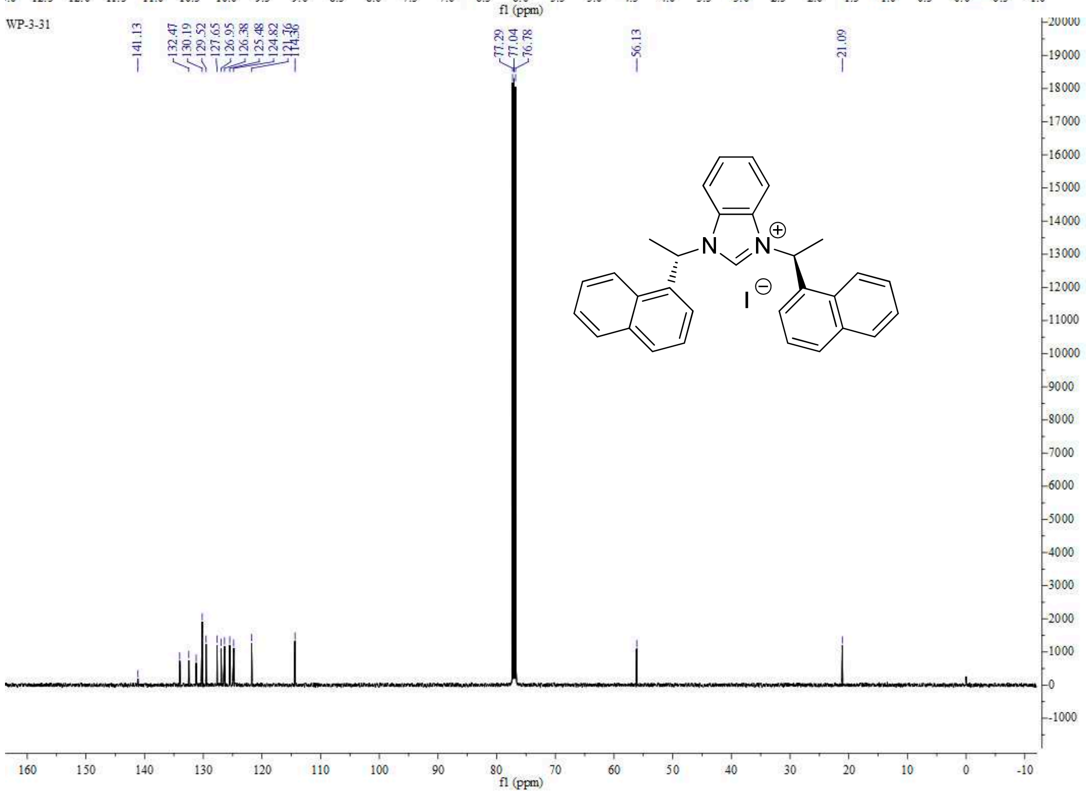
WP-3-31
141.13
132.47 130.19 129.52 127.65 126.95 126.38 125.48 124.82
77.29 77.04 76.78
56.13
21.09
N
N
I
f1 (ppm)

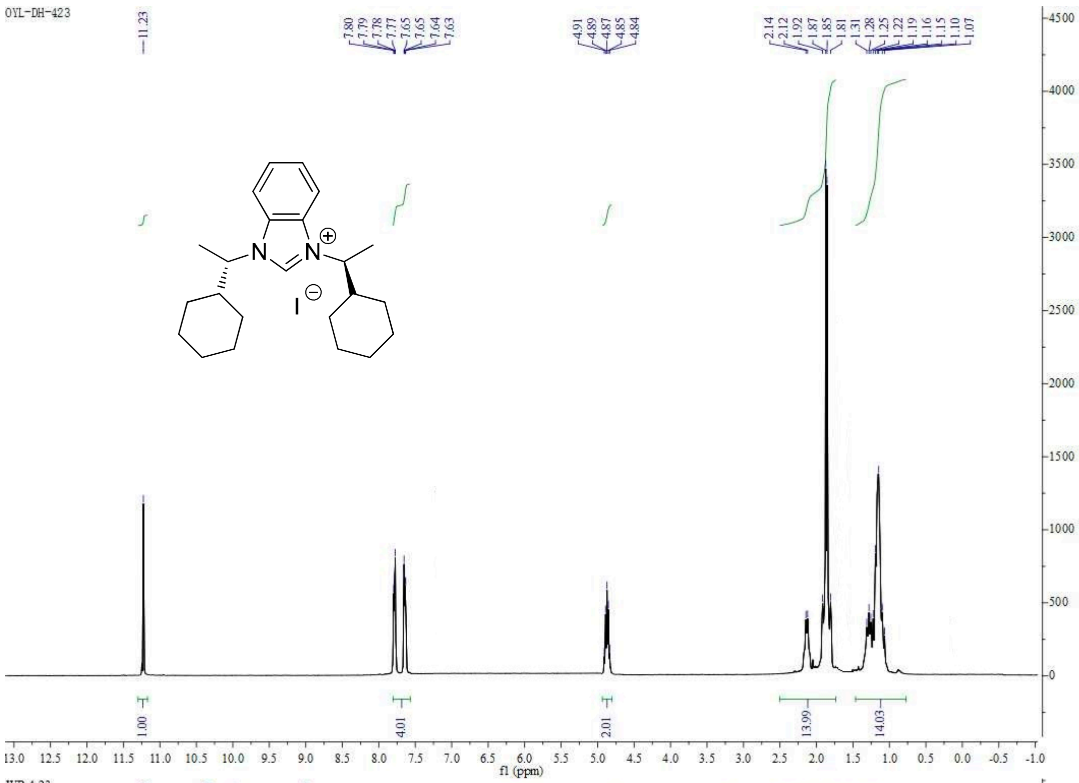
OYL-DH-423
11.23
7.80
7.79
7.78
7.77
7.65
7.65
7.64
7.63
4.91
4.89
4.87
4.85
4.84
2.14
2.12
1.92
1.87
1.85
1.81
1.31
1.28
1.25
1.22
1.19
1.16
1.15
1.10
1.07
1.00
4.01
2.01
13.99
14.03
f1 (ppm)

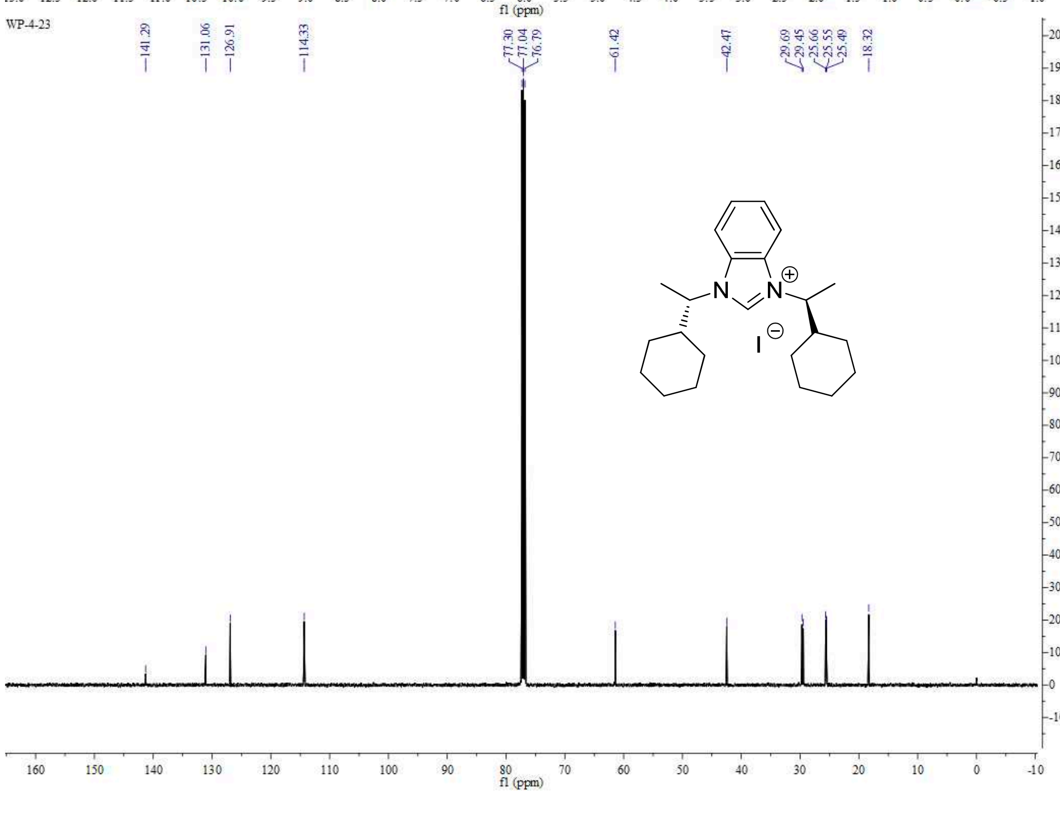
WP-4-23
141.29
131.06
126.91
114.33
77.30
77.04
76.79
61.42
42.47
29.69
29.45
25.66
25.55
25.49
18.32
f1 (ppm)

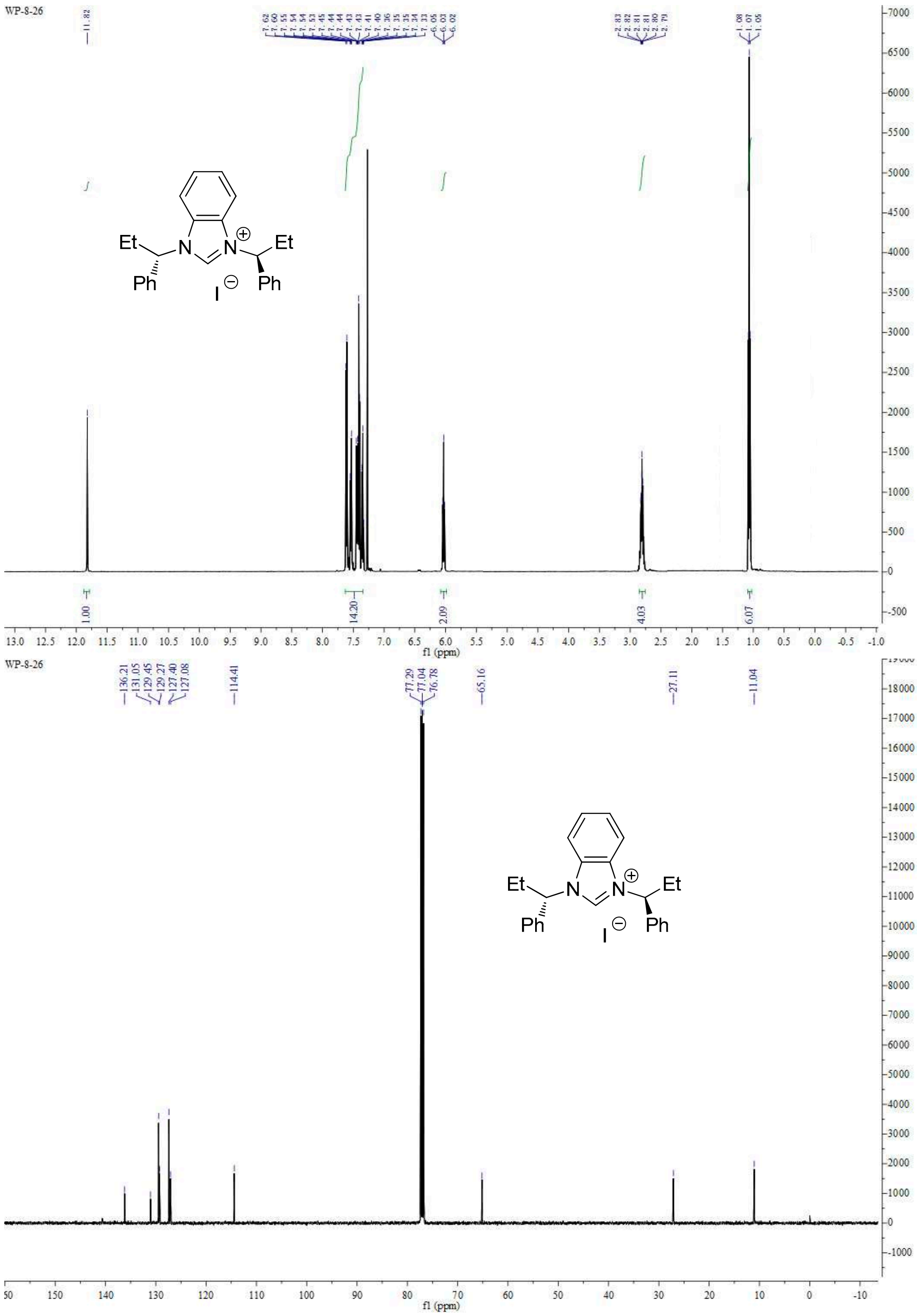

Figure S2. ^{1}H and ^{13}C-NMR Spectra of compounds **3a**–**d**.

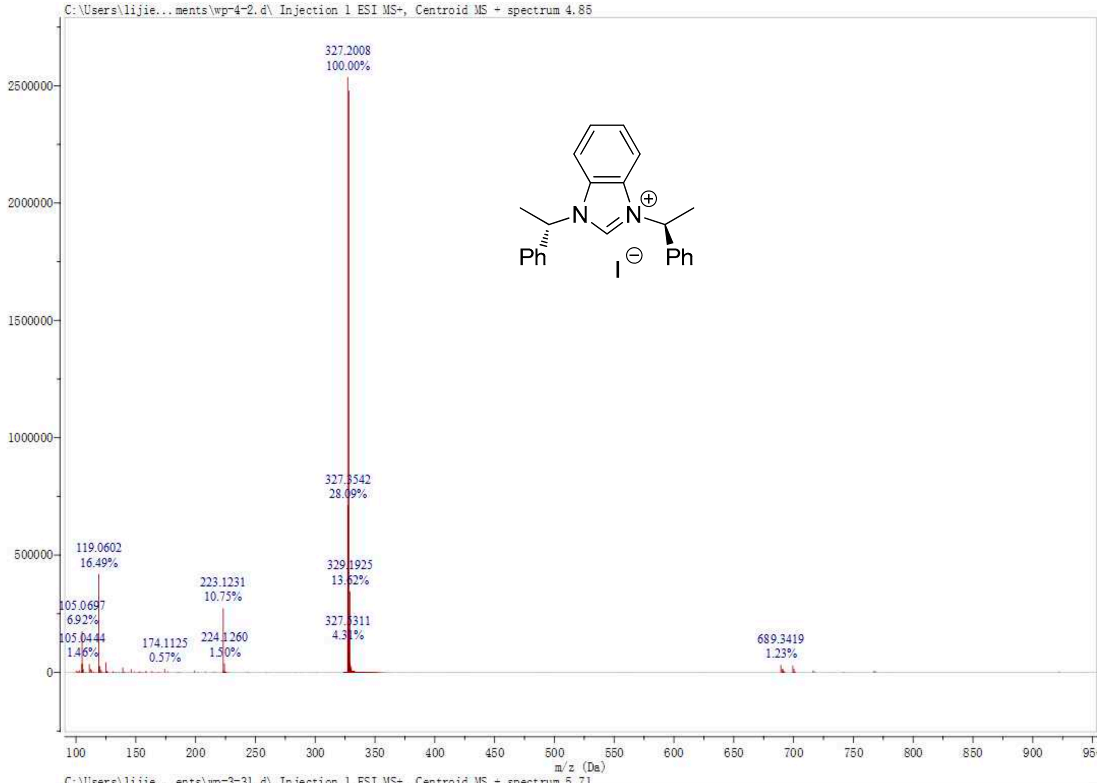
C:\Users\lijie... ments\wp-4-2.d\ Injection 1 ESI MS+, Centroid MS + spectrum 4.85
327.2008
100.00%
327.3542
28.09%
329.1925
13.62%
327.5311
4.31%
119.0602
16.49%
105.0697
6.92%
105.0444
1.46%
174.1125
0.57%
223.1231
10.75%
224.1260
1.50%
689.3419
1.23%
m/z (Da)
Ph
Ph
N
N
I

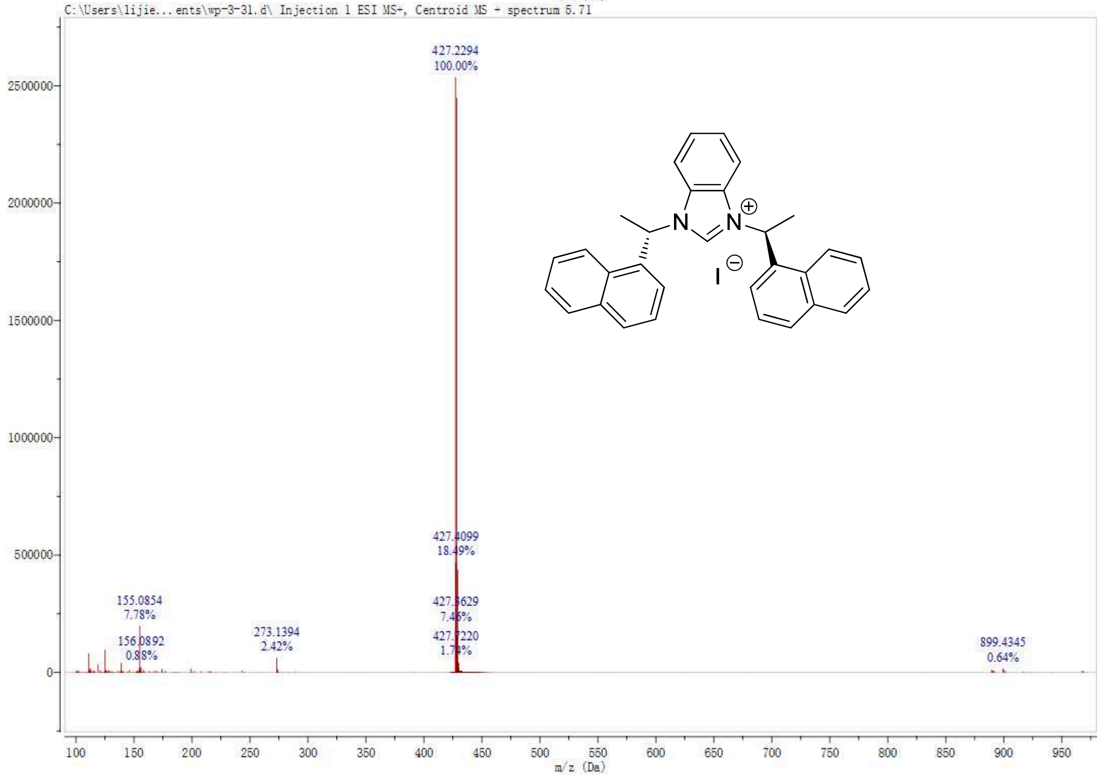
C:\Users\lijie... ents\wp-3-31.d\ Injection 1 ESI MS+, Centroid MS + spectrum 5.71
427.2294
100.00%
427.4099
18.49%
427.3629
7.46%
427.7220
1.74%
155.0854
7.78%
156.0892
0.88%
273.1394
2.42%
899.4345
0.64%
m/z (Da)
N
N
I

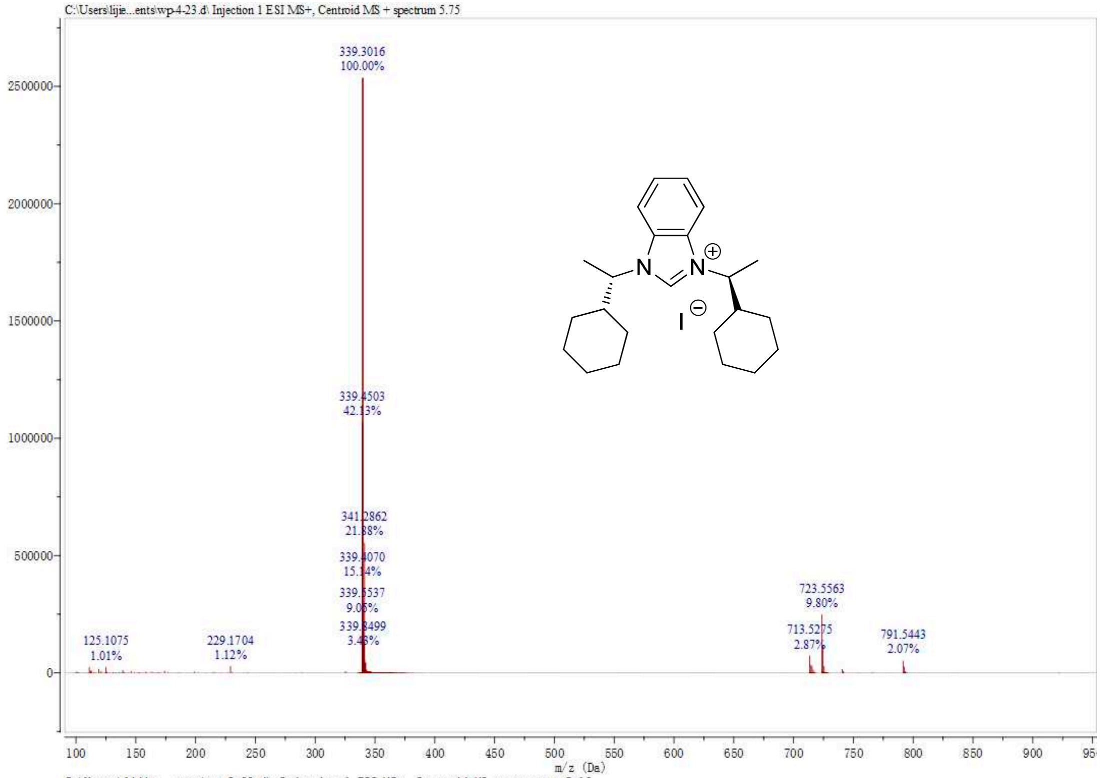

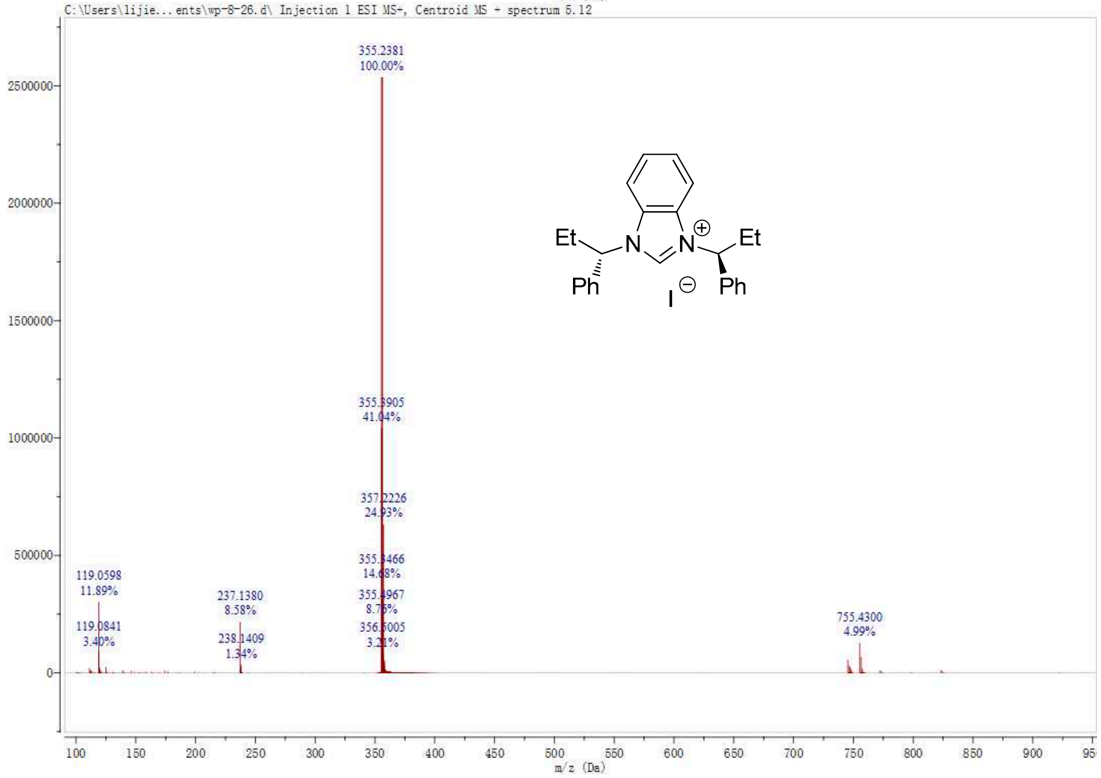

Figure S3. HR-MS Spectra for compounds **3a–d**.
